# Ventricular longitudinal function is associated with microvascular obstruction and intramyocardial haemorrhage

**DOI:** 10.1136/openhrt-2015-000337

**Published:** 2016-05-02

**Authors:** Pankaj Garg, Ananth Kidambi, James R J Foley, Tarique Al Musa, David P Ripley, Peter P Swoboda, Bara Erhayiem, Laura E Dobson, Adam K McDiarmid, John P Greenwood, Sven Plein

**Affiliations:** Multidisciplinary Cardiovascular Research Centre & Leeds Institute of Cardiovascular and Metabolic Medicine, University of Leeds, Leeds, UK

## Abstract

**Background:**

Microvascular obstruction (MVO) and intramyocardial haemorrhage (IMH) are associated with adverse prognosis, independently of infarct size after reperfused ST-elevation myocardial infarction (STEMI). Mitral annular plane systolic excursion (MAPSE) is a well-established parameter of longitudinal function on echocardiography.

**Objective:**

We aimed to investigate how acute MAPSE, assessed by a four-chamber cine-cardiovascular MR (CMR), is associated with MVO, IMH and convalescent left ventricular (LV) remodelling.

**Methods:**

54 consecutive patients underwent CMR at 3T (Intera CV, Philips Healthcare, Best, The Netherlands) within 3 days of reperfused STEMI. Cine, T2-weighted, T2* and late gadolinium enhancement (LGE) imaging were performed. Infarct and MVO extent were measured from LGE images. The presence of IMH was investigated by combined analysis of T2w and T2* images. Averaged-MAPSE (medial-MAPSE+lateral-MAPSE/2) was calculated from 4-chamber cine imaging.

**Results:**

44 patients completed the baseline scan and 38 patients completed 3-month scans. 26 (59%) patients had MVO and 25 (57%) patients had IMH. Presence of MVO and IMH were associated with lower averaged-MAPSE (11.7±0.4 mm vs 9.3±0.3 mm; p<0.001 and 11.8±0.4 mm vs 9.2±0.3 mm; p<0.001, respectively). IMH (β=−0.655, p<0.001) and MVO (β=−0.567, p<0.001) demonstrated a stronger correlation to MAPSE than other demographic and infarct characteristics. MAPSE ≤10.6 mm demonstrated 89% sensitivity and 72% specificity for the detection of MVO and 92% sensitivity and 74% specificity for IMH. LV remodelling in convalescence was not associated with MAPSE (AUC 0.62, 95% CI 0.44 to 0.77, p=0.22).

**Conclusions:**

Postreperfused STEMI, LV longitudinal function assessed by MAPSE can independently predict the presence of MVO and IMH.

**Key questions**

**What is already known about this subject?**

- Microvascular obstruction and intramyocardial haemorrhage are independent adverse prognostic markers after reperfused ST-elevation myocardial infarction.
- Both these pathological process affect predominantly the subendocardium.
- Subendocardial myocardial fibres are predominantly longitudinal and mainly contribute to the global left ventricular longitudinal function.
- Mitral annular plane systolic excursion (MAPSE) is a marker of left ventricular longitudinal function.

**What does this study add?**

- Left ventricular longitudinal function assessed by MAPSE is independently associated with microvascular obstruction and intramyocardial haemorrhage.
- Acute left ventricular longitudinal function does not demonstrate association with adverse left ventricular remodelling.
- A cut-off of 10.6 mm for averaged-MAPSE was 89% sensitive and 72% specific for the detection of microvascular obstruction and was 92% sensitive and 74% specific for the detection of intramyocardial haemorrhage.

**How might this impact on clinical practice?**

- MAPSE can be evaluated at bedside by routine echocardiography and is associated with the presence of prognostic bioimaging markers.
- High-risk patients identified with MAPSE may benefit from further more detailed imaging assessment.
- Further research is needed to evaluate how this patient group should be managed.

## Introduction

Following reperfused acute myocardial infarction (AMI), the left ventricle (LV) undergoes structural adaptations both within and outside of the infarct zone, referred to as LV remodelling. In approximately 30% of patients, coronary reperfusion is associated with microvascular obstruction (MVO).^1^ Reperfusion may also lead to intramyocardial haemorrhage (IMH) in the infarct core^2^ associated with extravasation of blood.^3^ MVO and IMH are associated with adverse prognosis and adverse LV remodelling, independently of infarct size.^4–6^

**Figure 1:**
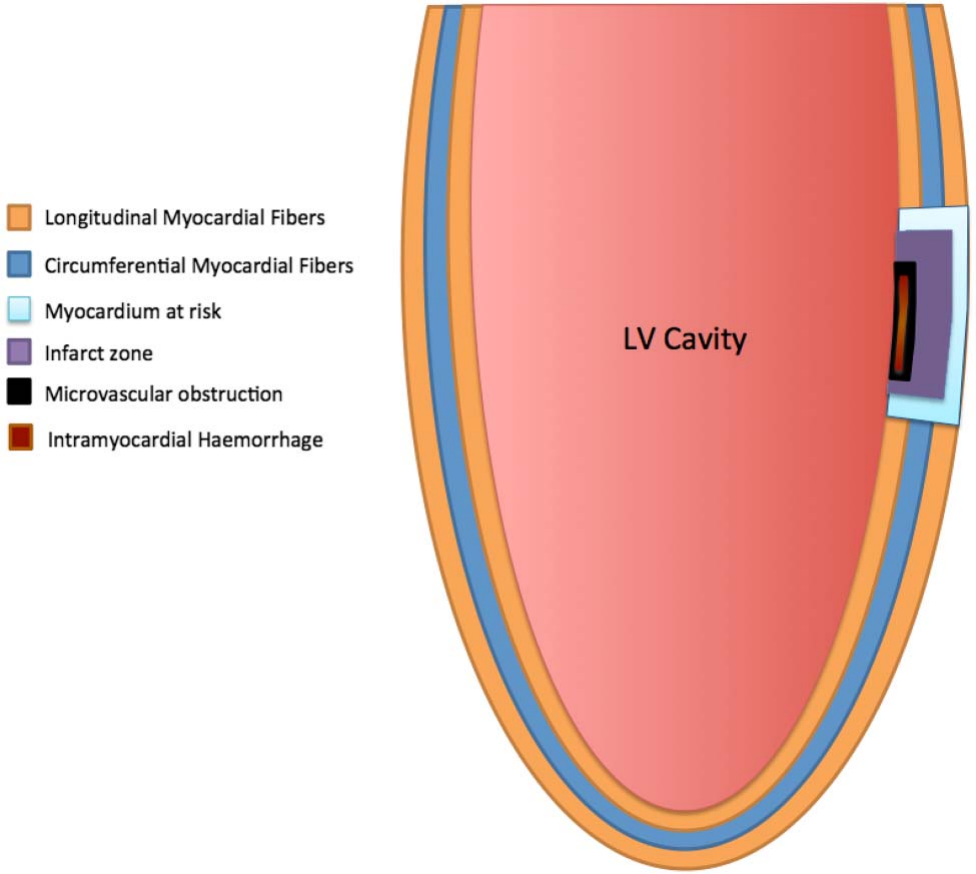
Illustration of orientation of myocardial fibres^9^ in long-axis view with representation of infarct related zones. LV, left ventricular.

Acute myocardial ischaemia initially affects the subendocardium and progresses to the subepicardial layers in a ‘wave front’ manner.^7^ ^8^ Similarly, MVO and IMH predominantly affect the subendocardial layer. Endocardial fibres are structurally longitudinal fibres^9^ and therefore predominantly contribute to longitudinal contractile function of the LV.^10^ MVO and IMH are therefore likely to affect predominantly longitudinal function^11^ (figure 1).

Mitral annular plane systolic excursion (MAPSE) is a well-established and easily obtained echocardiographic parameter for the assessment of longitudinal function and has been shown to correlate with LV systolic function.^12^ Post-AMI, MAPSE has prognostic importance in the risk stratification of patients, for example, a MAPSE of <8 mm is associated with a >3× higher incidence of hospitalisation and mortality (p=0.0001).^13^

Traditionally, longitudinal functional assessment using MAPSE has been based on M-mode transthoracic echocardiography. It can also be evaluated from a standard four-chamber cardiovascular MR (CMR) cine image. CMR-based MAPSE is easily measured, reproducible and strongly correlates with the echocardiographic equivalent.^14^ ^15^

We sought to investigate the effect of MVO and IMH on MAPSE in patients with acute STEMI and to determine correlations of CMR derived MAPSE with other markers of adverse outcome.

## Methods

### Patient selection

Patients presenting with first ST-segment elevation AMI who were revascularised by primary percutaneous coronary intervention (PPCI) within 12 h of onset of chest pain were prospectively recruited from a single tertiary centre. AMI was defined as per current guidelines.^16^ Exclusion criteria were previous AMI or coronary artery bypass grafting, cardiomyopathy, estimated glomerular filtration rate <30 mL/min/1.73 m^2^, haemodynamic instability or any contraindication to CMR. The study protocol was approved by the institutional research ethics committee and complied with the Declaration of Helsinki. All patients gave written informed consent to participate in this study. After PPCI, patients received standard post-AMI secondary prevention therapy and were enrolled in a cardiac rehabilitation programme.^17^

### Image acquisition

All patients had CMR imaging at 3.0T (Achieva TX, Philips Healthcare, Best, the Netherlands) within 3 days (median 2 days) of their index presentation. A dedicated 32-channel cardiac phased array receiver coil was used.

Cine imaging was performed using a balanced steady-state free precession (SSFP) pulse sequence with a spatial resolution of 1.6×2.0×10 mm and 40 phases per cardiac cycle. A four-chamber cine was planned perpendicular to the ventricular long axis and through the mitral annular plane and the LV apex.

T2w, T2* and late gadolinium enhancement (LGE) imaging were performed using the ‘3-of-5’ approach by acquiring the central 3 slices of 5 parallel short-axis slices spaced equally from mitral valve annulus to LV apical cap.^18^ 0.1 mmol/kg gadolinium-DTPA (gadopentetate dimeglumine; Magnevist, Bayer, Berlin, Germany) was then administered using a power injector (Spectris, Solaris, Pennsylvania, USA). LGE imaging was performed at 16–20 min following contrast administration. For each pulse sequence, images with artefact were repeated until any artefact was removed or minimised. The highest quality images were used for analysis.

### Image analysis

Cine, T2w, T2* and LGE images were evaluated offline using commercially available software (cvi42 v4.1.5, Circle Cardiovascular Imaging Inc., Calgary, Canada). Left ventricular volumes and ejection fraction (EF) were analysed from cine images using standard methods.^19^ Infarct location was determined by LGE imaging, according to standard guidelines.^20^ The presence and size of infarction and MVO were measured from LGE images. Infarct was defined as an area of LGE ≥2 SDs (SD) above remote myocardium, and infarct volume estimation included any hypointense core. We used the 2 SD method over the full-width halve maximum method as there is more prognostic data for the 2 SD infarct size estimation.^21^ The 2 SD cut-off was chosen for consistency with analysis of T2w images. MVO was defined visually as a hypointense core within the infarcted zone on LGE images and planimetered manually. Volumes of infarct and MVO were calculated from planimetered areas across the whole LV stack by the modified Simpson's method. The presence and extent of myocardial haemorrhage was assessed by combined analysis of T2w and T2* sequences.^8^ On T2w images, areas with mean signal intensity more than 2 SD below the periphery of the area at risk were considered to represent haemorrhage.^22^ On the T2* images, the presence of a dark core within the infarcted area by visual inspection of the images was used as confirmation of myocardial haemorrhage. Only when T2w and T2* images showed concordant findings was an area considered to represent haemorrhage. Presence of MVO and IMH were scored in a binominal mode.

**Figure 2:**
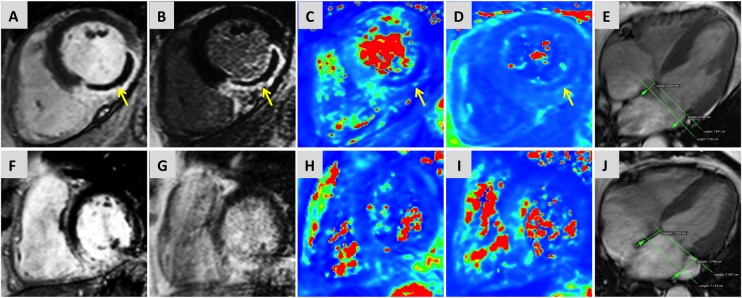
Multiparametric CMR examination of two cases of ST-elevation myocardial infarction. Case 1 (top row): Case of inferior-lateral MI with presence of MVO on early gadolinium enhancement imaging (A) and LGE (B). IMH within MVO was confirmed on T2-weighted maps (C) and T2*-maps (D). 4-chamber cine imaging (E) confirmed reduced averaged-MAPSE (overlay of systolic and diastolic annular position shown in green). Case 2: Case of inferior MI without the presence of MVO (F and G) or IMH (H and I) and comparatively better longitudinal displacement than case 1 (J). CMR, cardiovascular MR; IMH, intramyocardial haemorrhage; LGE, late gadolinium enhancement; MAPSE, mitral annular plane systolic excursion; MVO, microvascular obstruction.

For longitudinal functional assessment, the four-chamber cine images were used (figure 2). A region-of-interest (ROI) line was drawn across the medial and lateral mitral annulus as a reference point in end-diastole (just after closure of mitral valve). A second ROI line was drawn across the same plane on an image taken just after closure of the aortic valve, assessed from the LV outflow tract cine. The longitudinal distance between the two lines parallel to the left ventricular walls was measured for medial and lateral walls. For MAPSE, three parameters were assessed—septal wall MAPSE; lateral wall MAPSE and averaged-MAPSE (medial-MAPSE+lateral-MAPSE)/2).

Longitudinal functional assessment was carried out independently by two physicians experienced in both echocardiography and CMR imaging (PG, 5-year experience; JRJF, 4-year experience). For intraobserver variability assessment, one observer (PG) repeated the analysis after 90-days, blinded to the original results. Patients with suboptimal four-chamber cine images were excluded from analysis.

### Follow-up scans

Follow-up scans were planned at 3 months following the indexed event. For analysis, patients were divided into two groups based on the presence of LV remodelling, defined by the following: (A) an increase of LV end-diastolic volume >20% at 3 months' follow-up scan or (B) as increase of LV end-systolic volume >20% at 3 months' follow-up scan.

### Statistical analysis

Statistical analysis was performed using IBM SPSS Statistics V.21.0. Continuous variables are expressed as mean±SD. Normal distribution for quantitative data was established using the Kolmogorov-Smirnov test. Demographic comparisons were performed with an independent and paired two-sample t test. For follow-up data, the paired two-sample t test was used to investigate differences. Multivariate linear regression was used for variables with statistical significance from univariate analysis (p<0.1). The strength of a linear association between two variables was tested using Pearson's correlation coefficient (r). The accuracy of averaged-MAPSE in predicting presence of MVO and IMH was examined using receiver-operator characteristic curve analyses, using Medcalc (v14.12.0). Intra/interobserver variability was tested using coefficient of variation (CoV). All statistical tests were 2-tailed; p values <0.05 were considered significant.

## Results

Fifty four patients met the inclusion criteria. In five patients the infarct size was too small for accurate analysis; in another five patients the four-chamber cine was of insufficient quality for longitudinal functional analysis due to breathing artefact. Therefore 44 patients were included in the statistical analysis. Patient characteristics are shown in table 1. Infarct characteristics on CMR are listed in table 2. No gender-based differences in characteristics were present (p>0.1 for all).

**Table 1:** Patient characteristics

Patient characteristic	
n	44
Age, years	58.3±11.4
Male	37 (84%)
Body mass index, kg/m^2^	28.2±3.5
Current smoker	24 (55%)
Hypertension	11 (25%)
Hypercholesterolemia	13 (30%)
Diabetes mellitus	6 (14%)
Pain to balloon time, min (median (IQR))	213 (268)
TIMI flow grade 0 or 1 pre-PCI	40 (91%)
TIMI flow grade 3 post PCI	42 (95%)
Peak troponin I, ng/L (median)	>50 000
Peak CK, IU/L (median (IQR))	615 (1510)
Infarct territory	
Anterior	20 (45%)
Inferior	18 (41%)
Lateral	6 (14%)

Data as mean±SD or n (%) unless indicated.

CK, creatine kinase; PCI, percutaneous coronary intervention; PPCI, primary percutaneous coronary intervention; TIMI, flow grades based on results of the Thrombolysis In Myocardial Infarction trial.

**Table 2:** Infarct characteristics at baseline

Characteristic	First Scan
Ejection fraction, %	48±10
LV EDVi, mL/m^2^	82±16
LV ESVi, mL/m^2^	42±12
LV indexed mass, g/m^2^	65±14
LGE infarct volume, mL	15±12
LGE MVO volume, mL	3±5
MAPSE (septal)	9.6±2.9
MAPSE (lateral)	11±2.3
MAPSE (averaged)	10.3±2.1
MVO present	26 (59%)

n=44. Data as mean±SD. LV measurements are indexed to body surface area, infarct volumes are unindexed.

LGE, late gadolinium enhancement; LV, left ventricular; LV EDVi, left ventricular end diastolic volume (indexed); LV ESVi, left ventricular end systolic volume (indexed).

### Baseline data

The mean EF of the study cohort was 48±10%, septal MAPSE was 9.6±2.9 mm, lateral-MAPSE 11±2.3 mm and averaged-MAPSE of 10.3±2.1 mm.

**Figure 3:**
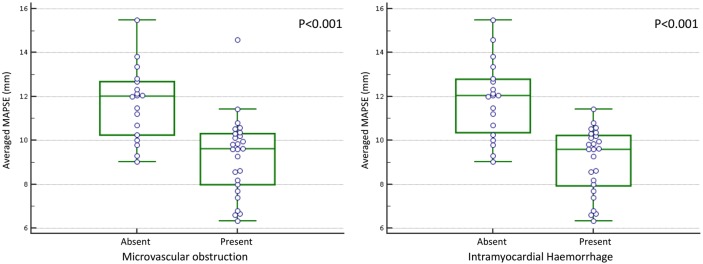
Box-plot of averaged-MAPSE with or without the presence of (A) MVO or (B) IMH. IMH, intramyocardial haemorrhage; MAPSE, mitral annular plane systolic excursion; MVO, microvascular obstruction.

**Figure 4:**
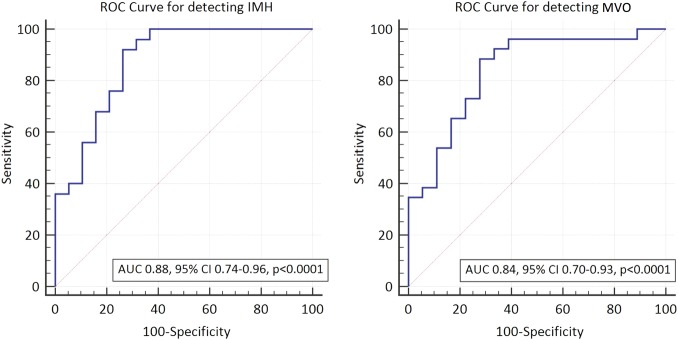
Receiver operator characteristics curve for the detection of (A) IMH, (B) MVO using averaged MAPSE. IMH, intramyocardial haemorrhage; MAPSE, mitral annular plane systolic excursion; MVO, microvascular obstruction.

Twenty-six (59%) patients had MVO and 25 had IMH. No patient had IMH without MVO. Averaged-MAPSE for patients with MVO was significantly lower than for patients without MVO (9.3±0.3 mm vs 11.7±0.4 mm; p<0.001). Similarly, in patients with IMH, MAPSE was significantly lower than in those without IMH (9.2±0.3 mm vs 11.8±0.4 mm; p<0.001) (figure 3). On linear regression analysis, using the variables in table 3, the presence of IMH (β= −0.65, p<0.001) and MVO (β=−0.57, p<0.001) demonstrated the strongest association with averaged-MAPSE (table 3). The size of MVO correlated negatively with averaged-MAPSE (r=−0.420, p=0.03). Infarct location did not influence medial-MAPSE (p=0.316), lateral-MAPSE (p=0.770) or averaged-MAPSE (p=0.391). The area under the curve (AUC) for determining the presence of MVO by averaged-MAPSE was 0.84 (95% CI 0.70 to 0.93; p<0.001) and for IMH was 0.88 (95% CI 0.77 to 0.96; p<0.001) (figure 4). The optimal cut-off value determined by the Youden index for averaged-MAPSE was 10.6 mm for the detection of MVO (sensitivity 88.5% and specificity 72.2%) and IMH (sensitivity 92.0% and specificity 73.7%).^23^

**Table 3:** Univariate and multivariate analysis of longitudinal parameters of LV function to CMR derived clinical and prognostic markers

	MAPSE (septal) p value		MAPSE (lateral) p value		MAPSE (averaged) p value (β)	
	UV	MV	UV	MV	UV	MV
Demographics						
Age	0.36		0.11		0.13	
Sex	0.27		0.44		0.74	
Smoking history	0.79		0.86		0.77	
Hypertension	0.64		0.65		0.94	
Hypercholesterolemia	0.81		0.69		0.95	
Diabetes mellitus	0.002	0.006	0.60		0.01	0.05
CK	0.26		0.72		0.33	
Pain-balloon time	0.0	0.004	0.26		0.45	
CMR parameters						
Anterior infarct	0.33		0.91		0.54	
LVEDVi	0.95		0.97		0.98	
SVi	0.06	0.187	0.27		0.06	0.207
Ejection fraction	0.02	0.246	0.09	0.82	0.01	0.293
Infarct mass	0.11		0.49		0.48	
Infarct volume	0.69		0.23		0.35	
MVO	0.002	0.415	0.001	0.36	<0.01 (−0.57)	0.229
IMH	<0.01	<0.01	<0.01	<0.01	<0.01 (−0.65)	<0.001

CK, creatine kinase; CMR, cardiovascular MR; IMH, intramyocardial haemorrhage; LV EDVi, left ventricular end diastolic volume (indexed); MAPSE, mitral annular plane systolic excursion; MV, multivariate; MVO, microvascular obstruction; SVi, systolic volume (indexed); UN, univariate.

### Follow-up data

Thirty-eight of 44 patients underwent day-90 CMR, while six patients refused follow-up scans. All 38 patients scans were of good quality and could be analysed. All patients had complete resolution of MVO and IMH on 90-day scans. Infarct volume reduced from 14.1±11.8 mLs at baseline to 8.2±6.7 mLs (p<0.001) at follow-up. As compared to baseline, EF improved by 21±22% and averaged-MAPSE improved by 28±22% (p<0.001 for each). On linear regression analysis taking into account day-2 CMR parameters (LVEDV, LVESV, averaged-MAPSE, MVO, IMH), LVESV at day-2 showed the strongest correlation with day-90 EF (r=0.717, p<0.001).

Of 38 patients with follow-up data, 9 patients (24%) demonstrated adverse left ventricular remodelling. LV remodelling showed no significant association with day-2 averaged-MAPSE (AUC 0.62, 95% CI 0.44 to 0.77, p=0.22) (figure 4C).

### Observer variability

On intraobserver analysis, the means of septal MAPSE (8.52±2.2 mm vs 8.54±2.1 mm; p=0.87, CoV 4%), lateral-MAPSE (9.97±2.2 mm vs 9.99±1.9 mm; p=0.92, CoV 5.9%) and septal MAPSE (9.24±1.8 mm vs 9.27±1.8 mm; p=0.88, CoV 4.1%) were similar.

On inter observer analysis, the means of septal MAPSE were (9.1±2.4 mm vs 9.3±2.2 mm; p=0.49, CoV of 8.3%), lateral-MAPSE (11.1±2.4 mm vs 11.5±2 mm; p=0.37, CoV 8.4%) and averaged-MAPSE (10.4±1.8 mm vs 10.1±1.8 mm; p=0.25, CoV 5.9%).

## Discussion

This study demonstrates that longitudinal LV function measured by averaged-MAPSE on CMR is strongly associated with the presence of MVO and IMH. Moreover, the size of MVO adversely affects averaged-MAPSE.

MAPSE is a well-established and endorsed parameter of global LV longitudinal function in echocardiography. A lower absolute value of MAPSE in post-AMI is a poor prognostic marker.^12^ Previous studies using speckle tracking echocardiography in AMI patients have shown that MVO determined on CMR adversely affects global longitudinal strain (GLS) on echocardiography.^24^ ^25^ However, with current technology, there remains marked variability in GLS depending on the measurement algorithms on the vendor's software and also on the sample volume definition. Therefore, no specific vendor independent normal ranges are provided in current American and European guidelines.^26^ MAPSE, on the contrary, is a vendor independent parameter of global longitudinal function, which can also be measured from standard cine CMR images.^27^ Limited data are available in the literature on the association of MAPSE assessed by CMR to MVO and none for IMH. This study demonstrated that reduced averaged-MAPSE is associated the presence of MVO/IMH.

Myocardial deformation, including global longitudinal function (GLS), can also be studied on CMR using strain measurements derived from tissue tagging or feature tracking (FT). Tagging derived strain requires longer scanning time and both methods need dedicated postprocessing software to analyse the data. In a post-AMI study comparing tagging to FT-derived strain, FT-derived strain was quicker to analyse, tracked myocardium better, had better interobserver variability and stronger correlations with infarct and oedema.^28^ Furthermore, similar to echocardiography, there remains significant intervendor and intersoftware variability in FT-derived strain.^29^ Hence, MAPSE remains the quickest, vendor-software independent and reproducible method to assess GLS from routinely acquired cine images.

Our data confirm previous observations of a weak association of LV remodelling with LV global longitudinal function,^30^ ^31^ due to the fact that longitudinal function of the LV is driven mainly by the endocardial fibres (figure 1). Also, longitudinal fibres contribute only 19% to the total stroke volume of the LV versus circumferential fibres which contribute 43%.^32^ Hence, even though MAPSE may be more adversely affected in patients with MVO or IMH, MAPSE does not necessarily result in LV remodelling. LV remodelling is directly proportional to the infarct transmural extent and hence more related to strain parameters, which involve more mid, and epicardial fibres, for example, circumferential strain.

### Clinical implications

Our findings have potential clinical implications and suggest that MAPSE can be performed easily on standard cine CMR images, without the need for additional MR tissue characterisation techniques (T2W and T2*) and analysis methods. As shown, MAPSE can potentially predict the presence of MVO/IMH early after primary PCI for STEMI. MVO and IMH are independent histopathological and cardiac imaging markers of adverse prognosis and their early detection from routinely acquired CMR images by MAPSE may help tailor appropriate pharmacological interventions. Patients with previous history of allergy to gadolinium-based contrast or patients with end-stage renal failure may also benefit from this technique to predict the likelihood of presence of MVO or IMH. MAPSE can also be evaluated by bedside M-Mode echocardiography in post infarct patients and it could potentially act as a gatekeeper for further assessment by CMR.

### Study limitations

The study sample size is relatively small and the results are therefore mainly hypothesis generating. In this study, we also excluded patients who were unstable post PPCI (higher Killip class, not able to lie flat because of shortness of breath and use of invasive monitoring). These patients are more likely to represent a higher risk group with more adverse prognosis. In our study population, the majority of patients with MVO had IMH and only one patient with MVO had no IMH. Hence, it was not possible to investigate whether there remains an incremental value of using averaged-MAPSE for the detection of IMH. Additionally, the absolute measure of MAPSE does not take the total length of the LV into account, which is potentially a better measure of LV longitudinal function as it measures absolute change in longitudinal parameters. In some diseases, like apical pericardial effusion, the apex may be mobile and this may influence the longitudinal function independently.

## Conclusions

Averaged-MAPSE, evaluated using cine CMR is strongly associated with the presence of MVO and IMH when compared to advanced MR relaxometry techniques (T2w and T2*), in patients with recent reperfused AMI. Global left ventricular longitudinal function, assessed by averaged-MAPSE, is feasible and shows high reproducibility. In this study, the size of MVO adversely affected averaged-MAPSE. However, averaged-MAPSE did not show any significant correlation with left ventricular remodelling.
